# Melatonin combats molecular terrorism at the mitochondrial level

**DOI:** 10.2478/v10102-010-0030-2

**Published:** 2010-11

**Authors:** Russel J. Reiter, Sergio D. Paredes, Ahmet Korkmaz, Mei-Jie Jou, Dun-Xian Tan

**Affiliations:** 1 The University of Texas Health Science Center, Department of Cellular and Structural Biology, San Antonio, Texas, USA; 2 Chang Gung University, Department of Physiology and Pharmacology, Kwei-Shan, Tao-Yuan, TAIWAN

**Keywords:** melatonin, mitochondria, free radicals, oxidative stress, mitochondrial complex inhibitors

## Abstract

The intracellular environmental is a hostile one. Free radicals and related oxygen and nitrogen-based oxidizing agents persistently pulverize and damage molecules in the vicinity of where they are formed. The mitochondria especially are subjected to frequent and abundant oxidative abuse. The carnage that is left in the wake of these oxygen and nitrogen-related reactants is referred to as oxidative damage or oxidative stress. When mitochondrial electron transport complex inhibitors are used, e.g., rotenone, 1-methyl-1-phenyl-1,2,3,6-tetrahydropyridine, 3-nitropropionic acid or cyanide, pandemonium breaks loose within mitochondria as electron leakage leads to the generation of massive amounts of free radicals and related toxicants. The resulting oxidative stress initiates a series of events that leads to cellular apoptosis. To alleviate mitochondrial destruction and the associated cellular implosion, the cell has at its disposal a variety of free radical scavengers and antioxidants. Among these are melatonin and its metabolites. While melatonin stimulates several antioxidative enzymes it, as well as its metabolites (cyclic 3-hydroxymelatonin, N^1^-acetyl-N^2^-formyl-5-methoxykynuramine and N^1^-acetyl-5-methoxykynuramine), likewise effectively neutralize free radicals. The resulting cascade of reactions greatly magnifies melatonin's efficacy in reducing oxidative stress and apoptosis even in the presence of mitochondrial electron transport inhibitors. The actions of melatonin at the mitochondrial level are a consequence of melatonin and/or any of its metabolites. Thus, the molecular terrorism meted out by reactive oxygen and nitrogen species is held in check by melatonin and its derivatives.

## Introduction

Melatonin (*N*-acetyl-5-methoxytryptamine) is an endogenously-produced molecule found throughout the animal kingdom from unicells to humans (Reiter, 1991a; Hardeland and Poeggeler, 2003). It is also present in plants (Hattori *et al*., 1995; Reiter *et al*., 2007; Kolar and MachacKova, 2005). Within multicellular organisms melatonin is generated in a wide variety of different tissues, although the pineal gland is the best known source of the indoleamine in vertebrates (Quay, 1974; Reiter, 1991b). Other major sites of melatonin production include the eye (Tosini *et al*., 2006; Itoh *et al*., 2007), brain (Jimenez-Jorge *et al*., 2007), gut (Bubenik and Pang, 2007), bone marrow (Tan *et al*., 1999a), skin (Slominski *et al*., 2005), immune cells (Carrillo-Vico *et al*., 2005) and others (Stefulj *et al*., 2001). In mammals, melatonin in the blood is primarily derived from the pineal gland (Quay, 1974; Reiter, 1991a). In the blood, melatonin levels exhibit a circadian rhythm with highest concentrations occurring during the dark period, coincident with maximal nocturnal melatonin production in the pineal gland (Reiter, 1987; 1991a). A melatonin rhythm similar to that in the blood also occurs in the cerebrospinal fluid (Skinner and Malpaux, 1999), saliva (Laakso *et al*., 1990) and at least one of its metabolites, 6-hydroxymelatonin, in the urine (Cavallo and Dolan, 1996).

Light-at-night quickly depresses pineal melatonin production via a pathway that involves a unique photopigment, melanopsin, in specialized photoreceptive retinal ganglion cells in the lateral eyes (Panda *et al*., 2005). The ability of light to inhibit nocturnal melatonin synthesis is both intensity (McIntytre *et al*., 1989) and wavelength (Brainard *et al*., 2001) dependent. As pineal melatonin synthesis falls due to nocturnal light exposure, likewise blood melatonin levels drop (Reiter, 1991a).

Besides light-at-night, a second major feature that reduces melatonin formation is age. In all species examined to date, increasing age is associated with a gradual waning of the nocturnal melatonin rhythm such that the elderly may be almost devoid of this important molecule (Reiter *et al*., 1980, 1981; Sack *et al*., 1986). Given the marked beneficial actions of melatonin at the cellular level, particularly in mitochondria (Acuna-Castroviejo *et al*., 2001; Leon *et al*., 2004, 2005), the gradual reduction of melatonin throughout life may contribute to the persistent accumulation of oxidatively-modified molecules and worsening of a number of mitochondria-related disorders (Acuna-Castroviejo *et al*., 2002; DiMauro and Schon, 2003).

Melatonin is a ubiquitously-acting molecule which can function as an autocoid, paracoid, hormone, antioxidant and as a tissue factor (Tan *et al*., 2003). As such, this indoleamine mediates changes in seasonal reproduction in photoperiodic species (Reiter, 1980; Goldman, 1999), influences retinal physiology (Morgan and Boelan, 1996; Tosini, 2000), modulates the immune system (Guerrero and Reiter, 2002; Carrillo-Vico *et al*., 2006), inhibits the growth of a number of cancer cells (Blask *et al*., 2005; Leon-Blanco *et al*., 2003; Sanchez-Barcello *et al*., 2005), reduces oxidative stress (Siu *et al*., 2006; Maldonado *et al*., 2007; Jaworek *et al*., 2005), promotes sleep (Zisapel, 2007; Jan *et al*., 2007) and others.

Mechanistically, melatonin achieves its actions via a number of means. Many cells are equipped with membrane receptors which allow them to respond to the circadian melatonin message (Dubocovich and Markowska, 2005; Witt-Enderby *et al*., 2006). Melatonin binding sites (receptors) have also been found in the nuclei of many cells (Pozo *et al*., 2003; Coto-Montes *et al*., 2003). Whereas the signal transduction mechanisms involved in the actions of melatonin at the level of the membrane receptors have been reasonably well defined, information on these processes in terms of the nuclear binding sites remain, for the most part, enigmatic. Intracellularly, melatonin also binds to calmodulin allowing it to regulate several intracellular events, e.g., nitric oxide synthase (NOS) activity (Pozo *et al*., 1997; Benitez-King, 2006).

Additionally, melatonin has actions that do not require a receptor or formation of a complex with another molecule. This receptor-independent action, i.e., radical scavenging, only requires that melatonin be in the vicinity of a reactive oxygen or nitrogen specie when it is generated (Reiter, 1995; Tan *et al*., 2002; Hardeland *et al*., 2003a). Given that mitochondria are a major site of free radical production, it would obviously be advantageous if the concentrations of melatonin were also high within mitochondria. Whereas some preliminary data suggest that this organelle may contain melatonin concentrations that exceed levels in the blood or other portions of the cell (Martin *et al*., 2000), this evidence is certainly not unassailable.

## Mitochondria and free radical generation

The inner mitochondrial membrane is unusually rich in proteins, half of which are involved in electron transport and in oxidative phosphorylation. The electron transport chain (ETC) which is coupled to oxidative phosphorylation provides cells with their major means of generation of its energy requirements (Scheffler, 1999). Up to 95% of the ATP produced in aerobic cells is a result of mitochondrial oxidative phosphorylation. The activities of the ETC and oxidative phosphorylation must remain continuously active to ensure mitochondrial and cellular survival.

The majority of molecular oxygen (O_2_) inhaled and eventually taken up by cells is processed in the mitochondrial ETC where it is converted to water following its four electron reduction. However, during this reductive process, partially reduced species of O_2_ are also produced including reactants that are reduced by one, two or three electrons, i.e., the superoxide anion (O_2_·^–^) and hydroxyl radical (·OH) and one non-radical product, hydrogen peroxide (H_2_O_2_). Collectively, these agents are referred to as reactive oxygen species (ROS).

Free radicals generally have the capability to pair up their unpaired electron by abstracting one from another molecule (thereby damaging it). O_2_ ·^–^ is not especially reactive in this regard. When formed, O_2_·^–^ is usually quickly dismutated to H_2_O_2_ by cytosolic or mitochondrial superoxide dismutase (CuZnSOD and MnSOD), respectively. Since H_2_O_2_ is the immediate precursor of the highly damaging ·OH, it is imperative that H_2_O_2_ be removed from the intramitochondrial environment as quickly as possible. The major enzyme that accomplishes this is glutathione peroxidase (GPx), which metabolizes H_2_O_2_ to water and O_2_; in this process GPx also converts reduced glutathione (GSH) to its oxidized metabolite (GSSG). Given the major importance of GSH in mitochondrial physiology, it is essential that GSSG be reduced back to GSH; this is accomplished by glutathione reductase (GRd) (Fernandez-Checa and Kaplowski, 2005).

The removal of H_2_O_2_ from the mitochondrial environment is never complete and, via the Fenton reaction, some damaging ·OH are always formed. Cellular organelles have no enzymatic means to remove ·OH so it must either be neutralized by a free radical scavenger or it mutilates a bystander molecule. This carnage occurs in the immediate vicinity of where the ·OH is formed because of its extremely rapid reaction rate; the damage is referred to as being site specific.

In addition to ROS, mitochondria also generate reactive nitrogen species (RNS); two of these are nitric oxide (NO) and the peroxynitrite anion (ONOO^–^). Mitochondrial NO functions as a reversible antagonist of complex IV of the ETC by competing with O_2_ for its binding site. Usually tissue concentrations of NO and O_2_ are, respectively, in the ranges of 100–500 nM and 10–30 µM. In these concentration ranges, NO causes roughly half maximal inhibition of mitochondrial respiration. Thus, NO is a physiological regulator of respiration and also of the rate of ATP synthesis (Brown, 1992). Elevation of NO levels higher than the concentrations mentioned above has the potential of inhibiting not only complex IV but also complexes I and III. In this eventuality, mitochondrial electron transfer reactions are compromised and electron leakage is exaggerated leading to increased formation of O_2_·^–^ and all down stream oxidants (Brown and Borutaite, 2004).

Additionally, NO readily couples with the O_2_·^–^ to produce the non-radical species, the ONOO^–^, which irreversibly damages respiratory complexes (Cadenas *et al*., 1996). The toxicity of the ONOO^–^ is probably on a par with that of the ·OH with the resultant damage potentially causing mitochondrial dysfunction and cell death (Brown, 1992).

A specific isoform of nitric oxide synthase (NOS) has been proposed to exist in mitochondria (mtNOS) (Ghafourifar and Cadenas, 2005). This constitutively expressed enzyme is believed to derive from a neuronal NOS isoform (Tatoyan and Giulivi, 1998). NO produced within mitochondria under normal conditions may serve a regulatory purpose, but when generated in excess, such as during inflammation (Escames *et al*., 2006), it may inhibit respiration and ATP production. Melatonin has been shown to inhibit NOS (Leon *et al*., 2004; 2005; Maldonado *et al*., 2007).

## Melatonin as a free radical scavenger and as an antioxidant

Melatonin was discovered to be a free radical scavenger in 1993 (Tan *et al*., 1993). In this capacity, it is unexpectedly highly effective. Using the most definitive means of identifying a scavenger, i.e., a spin trapping agent and electron spin resonance spectroscopy, melatonin was documented to be a potent scavenger of the devastatingly reactive ·OH. This seminal observation has been repeatedly confirmed in pure chemical systems in both in vitro and in vivo studies (Matuszek *et al*., 1997; Bromme *et al*., 2000; Li *et al*., 2002; Sofic *et al*., 2005; Valko *et al*., 2005; Fukutomi *et al*., 2006). Invariably, the degree of molecular damage that occurs as a consequence of ·OH generation is reduced in the presence of melatonin. This is obvious in the indoleamine's ability to protect against ionizing radiation, a process known to produce ·OH in excess (Vijayalaxmi *et al*., 1999; Sever *et al*., 2004; Zhou *et al*., 2006).

Actually, melatonin scavenges two ·OH and, in the process, it is converted to cyclic 3-hydroxymelatonin (3-OHMEL). This latter product was identified by mass spectral analysis and carbon and proton-nuclear magnetic resonance (Tan *et al*., 1998; 1999b). Small amounts of cyclic 3-OHMEL are excreted in the urine (Ma *et al*., 2006); this also indicates that melatonin scavenges the ·OH in vivo. Cyclic 3-OHMEL is also formed during the interaction of melatonin with ONOO^–^ (Zhang *et al*., 1999; Peyrot *et al*., 2002). Although cyclic 3-OHMEL is not commercially available, a method for its synthesis has been published (Sawicka *et al*., 2004) for those interested in using this molecule.

Like its parent molecule, cyclic 3-OHMEL is also a highly effective free radical scavenger and, in doing so, it is converted to N^1^-acetyl-N^2^-formyl-5-methoxykynuramine (AFMK) (Tan *et al*., 2000). This latter molecule is interesting and its existence has been known for more than 30 years after its discovery in the rat brain, where it is enzymatically formed by indoleamine 2,3-dioxygenase from melatonin (Hirata *et al*., 1974). For decades this was thought to be the exclusive means of AFMK formation in vivo. It is now known, however, that it may be non-enzymatically produced when melatonin scavenges H_2_O_2_ (Tan *et al*., 2000), as well as when it interacts with O_2_ ·^–^ (Hardeland *et al*., 1995), singlet oxygen (^1^O_2_) (de Almeida *et al*., 2003), or the ONOO^–^ (Hardeland *et al*., 2003b). Finally, activated neutrophils and macrophages oxidize melatonin to AFMK (Silva *et al*., 2004). In plants as well as in animals, AFMK may be a major oxidation production of melatonin (Tan *et al*., 2007a). Collectively, the published findings suggest that AFMK is widely distributed in the animal and plant kingdoms. Like other melatonin catabolites, AFMK is found in the rat urine. Tan and colleagues (2007b) have speculated that AFMK may be a major derivative of melatonin.

AFMK has the ability to interact with the ABTS cation radical as well as with ROS/RNS to form N^1^-acetyl-5-methoxykynurmaine (AMK) (Rosen *et al*., 2006). AMK is then both a secondary or tertiary metabolite of melatonin. When AMK interacts with the ABTS cation radical (Than *et al*., 2006) or with ONOO^–^ (Guenther *et al*., 2005) it forms products that may also be ROS and RNS scavengers.

**Figure 1:**
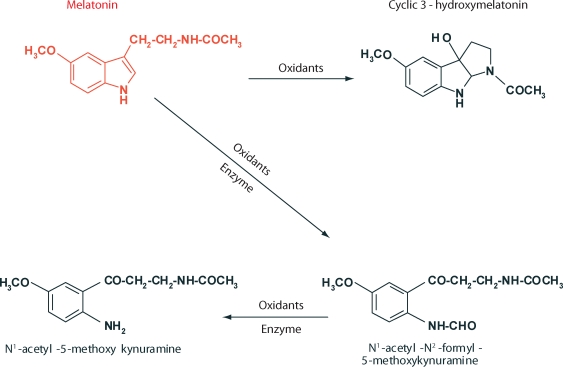
Melatonin oxidation and the products formed. While the parent molecule, melatonin, is a potent free radical scavenger, so are its metabolites. This scheme is referred to as the scavenger cascade of melatonin. It has been calculated that one molecule of melatonin may scavenge up to ten ROS/RNS. The figure is a composite of the findings published by Hardeland and colleagues (1995; 2003a, 2003b) and Tan *et al*. (1993; 2000).

This sequential scavenging of toxic reactants by melatonin and its metabolites is referred to as its antioxidative cascade and it is believed to be functional in vivo (Tan *et al*., 2007a). In this scheme it has been estimated that a single molecule of melatonin may neutralize up to ten toxic reactants. The oxidation reactions of melatonin as summarized in the foregoing paragraphs are summarized in Figure 1.

Supplementing the antioxidant capacity of melatonin is the ability of the indoleamine to promote the metabolism of toxic reactants to innocuous molecules. A number of antioxidative enzymes are important in limiting the oxidative burden of cells and organisms. As noted above, the major enzymes in this category are CuZnSOD, MnSOD, catalase (CAT), GPx and GRd. The SOD isoforms dismutate O_2_ ·^–^ to H_2_O_2_ at different sites within the cell, i.e., CuZnSOD works in the cytosol while MnSOD is confined to mitochondria. While H_2_O_2_ is not very reactive it has a long half-life and can pass through cell membranes easily and, due to its conversion to the ·OH via the Fenton reaction, it can cause molecular damage at sites distant from its place of origin. To prevent this from happening, it is catalytically removed from the cellular environment by GPx and CAT. When H_2_O_2_ and/or other hydroperoxides are metabolized by GPx, GSH is likewise converted to its oxidized form, GSSG. Since it is important that a very high percentage of the glutathione in a cell be in the reduced form, GSSG is quickly metabolized back to GSH by the enzyme GRd.

It has been known for more than a decade that the activities of the antioxidative enzymes mentioned above are heightened in the presence of exogenously administered, pharmacological doses of melatonin (Barlow-Walden *et al*., 1995; Pablos *et al*., 1995). Furthermore, the physiological rise in circulating levels of melatonin at night reportedly enhances antioxidative enzyme activities (Pablos *et al*., 1998; Baydas *et al*., 2002). Besides seemingly inciting these enzymes, in some cases melatonin may merely protect these proteins from oxidative damage and thereby preserve their function.

Two recent reviews have considered the mechanisms involved in stimulation of antioxidative enzyme activities by melatonin (Rodriguez *et al*., 2004; Tomas-Zapico and Coto-Montes, 2005). While the direct free radical scavenging actions of melatonin described are obviously receptor-independent, there is a general consensus, although the evidence is not incontrovertible, that the induction of these enzymes by melatonin likely involves receptors. The proposed mechanisms include an interaction of melatonin with both membrane receptors and nuclear binding sites. The fact that melatonin (and its metabolites) functions in the direct scavenging of ROS/RNS as well as activating enzymes which remove these potentially toxic agents from the intracellular environment greatly increases its ability to ward off molecular damage. Whereas melatonin acts as a direct free-radical scavenger in every cell, its stimulation of antioxidative enzymes would be limited to cells that possess receptors for the indoleamine, if the theory presented by Rodriguez *et al*. (2004) and Tomas-Zapico and Coto-Montes (2005) is valid. Considering the very wide spread distribution of at least the membrane melatonin receptors (Dubocovich and Markowska, 2005), and also presumably the nuclear binding sites (Pozo *et al*., 2003; Coto-Montes *et al*., 2003; Carrillo-Vico *et al*., 2005), this action of melatonin may also occur essentially throughout the organism.

NOS, of which there are several isoforms, is the rate-limiting enzyme in the production of the gaseous neurotransmitter, NO. NO has both highly beneficial as well as negative consequences in organisms. As mentioned previously, this nitrogen-based reactant quickly couples with O_2_·^–^ to form ONOO^–^ which is highly reactive and destructive. Thus, NOS is often considered a pro-oxidative enzyme and its inhibition leads to reduced oxidative damage both at the cytosol and mitochondrial levels (Reiter *et al*., 2000; Acuna-Castroviejo *et al*., 2005). Melatonin reduces the activity of this enzyme when administered to experimental animals and at the same time it lessens free radical mutilation of essential molecules (Pozo *et al*., 1997; Benitez-King, 2006; Escames *et al*., 2004; Leon *et al*., 2006). In some cases, the inhibition of NOS by melatonin may contribute significantly to the total antioxidative capacity of the indoleamine particularly at the mitochondrial level (Acuna-Castroviejo *et al*., 2005).

As already noted, keeping GSH levels high within cells is an important means of preserving the morphological and functional activities of molecules which would otherwise be degraded by free radicals and associated reactive products. In 1999, Urata and colleagues specifically tested whether melatonin would preserve intracellular GSH concentrations by enhancing its synthesis. They found, in fact, that melatonin stimulated the activity of the rate-limiting enzyme, γ-glutamylcysteine synthase (γ-GCS), in the production of this tripeptide antioxidant. Often, under elevated oxidative stress conditions, melatonin preserves intracellular GSH levels. This is not necessarily related to the ability of melatonin to stimulate γ-GCS since the indoleamine could preferentially scavenger free radicals and thereby preserve basal intracellular GSH concentrations. The stimulation of γ-GCS, as originally described by Urata *et al*. (1999), has been confirmed (Winiarska *et al*., 2006).

One final aspect should be considered when melatonin's ability to attenuate molecular impairment due to ROS/RNS is discussed. It is what Hardeland (2005) refers to as “radical avoidance”. Melatonin has stimulatory effects particularly on complexes I and IV of the mitochondrial ETC; as such, the number of electrons that leak from the complexes is reduced and free radical generation is attenuated. Limited free radical generation would result in less harm to neighboring molecules.

This section briefly summarizes the multiple processes by which melatonin may restrict the destruction of molecules and organelles normally inflicted by ROS/RNS. Whereas these processes have all been documented in vivo, the significance of each in forestalling oxidative damage may be cell specific.

## Mitochondrial toxins: protection by melatonin

Several drugs have been identified which are classified as mitochondrial poisons, i.e., they interfere with the transfer of electrons through the ETC. These drugs greatly exaggerate the escape of electrons into the mitochondrial intramembraneous space leading to a reduction of molecular oxygen and formation of radicals. Additionally, reduced oxidative phosphorylation precipitates a depletion of ATP (Beal, 1992). These changes cause elevated oxidative injury and an energy deficiency which, in the worst cases, leads to cellular and organismal death. The efficacy of melatonin in reducing the toxicity of some mitochondrial poisons has been examined.

### Rotenone

Rotenone, a specific inhibitor of complex I of the mitochondrial ETC, causes the generation of an excessive number of free radicals. The damage inflicted by this drug is believed to be a consequence of the ·OH (Saravanan *et al*., 2005, 2006). Rotenone has been used to induce a neurodegenerative condition in animals that is reminiscent of Parkinsonism in humans since its action in complex inhibition is considered specific to dopamine-containing neurons. The classic anti-Parkinson drug, selegiline, protects against the neurodegenerative changes that are a consequence of rotenone due to its ·OH scavenging actions (Saravanan *et al*., 2006).

Saravanan *et al*. (2007) stereotoxically infused rotenone (6µg in 1 µL) unilaterally into the right substantia nigra (SN) of rats to induce a condition of hemiparkinsonism and tested the effect of melatonin (10, 20 or 30 mg/kg, i.p., 30 minutes before surgery and at 12 hour intervals after surgery for 4 days) in modulating the oxidative changes. At the termination of the study (5 days), measures of oxidative stress were compared in the infused and contralateral (non-rotenone treated) brain regions. On the rotenone-infused side of the brain nigral GSH levels were depressed by 49% while in the area of the terminals of the dopaminergic neurons (caudate nucleus, putamen; CNP), the reduction was 26%. Melatonin at all doses significantly reduced the loss of GSH in the SN while the two larger doses also limited the depletion of GSH in the CNP. When the rotenone-infused SN was compared with its counterpart on the contralateral side of the brain (neural tissue obtained by micropunch), the drug increased both cytosolic SOD and CAT activities in the drug-damaged SN; the activities of both enzymes were further augmented after melatonin administration. The elevated enzyme activities after rotenone infusion were considered a compensatory response to the elevated oxidative stress. The additional rises induced by melatonin are consistent with its frequently-reported stimulation of antioxidative enzyme activities (Rodriguez *et al*., 2004; Tomas-Zapico and Coto-Montes, 2005). That melatonin limited the loss of GSH in the rotenone-infused SN and associated CNP was explained by the fact that this indoleamine stimulates the rate limiting enzyme, γ-GCS, for the synthesis of this tripeptide antioxidant (Urata *et al*., 1999; Winiarska *et al*., 2006).

In an important ancillary study published in the same report, Saravanan and co-workers (2007) incubated neurally-derived submitochondrial particles (P2 fraction) with rotenone (100 µM), salicylate (0.7 mM) and different concentrations of melatonin (0.1–1,000 µM) for 30 minutes. The formation of ·OH adducts of salicylate, i.e., 2,3-dihydroxybenzoic acid and 2,5-dihydroxybenzoic acid (2,3-DHBA and 2,5-DHBA, respectively) were estimated employing HPLC-electrochemistry. The addition of rotenone to the mitochondrial particles caused very large increases in 2,3-DHBA and 2,5-DHBA, indicative of highly elevated ·OH generation, while melatonin significantly attenuated rotenone-mediated ·OH formation by the mitochondria. The ability of melatonin to reduce ·OH-salicylate adducts was not unexpected considering that melatonin, as well as its metabolites, are highly efficient scavengers of not only the ·OH but any other radical or reactive species that may have been produced as a result of rotenone treatment (Tan *et al*., 1993, 2007b; Stasica *et al*., 1998, Hardeland, 2005).

Incubating isolated rat brain mitochondria with rotenone and Ca^2+^ induces marked oxidative damage to this organelle. The addition of melatonin to the purified mitochondria reduced the oxidative mutilation caused by the combination of rotenone and Ca^2+^ (Sousa and Castillo, 2005). Also, the addition of a Ca^2+^ ionophore (A23187), strongly potentiated rotenone-mediated death of pheochromocytoma (PC12) cells, a response attenuated by melatonin. Furthermore, in the presence of melatonin, free radicals were not detected to be released from PC12 cells co-exposed to rotenone plus the Ca^2+^ ionophore. Since melatonin did not change the concentration of Ca^2+^ nor did it prevent the inhibitory effect of rotenone on mitochondrial complex I, the authors concluded that the beneficial effects of melatonin on the mitochondria were primarily related to the antioxidative and free radical scavenging capacity of the indoleamine (Sousa and Castillo, 2005).

The fruit fly, *Drosophila melanogaster*, has been developed as a model for several neurodegenerative diseases, including Parkinsonism (Feany and Bender, 2000; Auluck *et al*., 2002). This model was used to examine the ability of melatonin to change the response of flies to rotenone treatment (Coulom and Birman, 2004). The mitochondrial toxin was fed to flies in the food they consumed; likewise, melatonin was administered via this route. Within a week, rotenone-only fed flies exhibited characteristic motor deficits and selective degeneration of the dopamine-containing neurons. When *Drosophila* were co-exposed to rotenone plus melatonin there was a markedly preserved motor behavior of the flies and, furthermore, melatonin was clearly superior to L-dopa in improving the score of the flies in the geotaxis test. When the number of dopamine-containing cells in the nervous system was compared in rotenone-treated flies with and without melatonin, it was obvious that the indoleamine had spared the dopaminergic neurons in many of the neuronal clusters from degeneration. In the rotenone-treated flies given melatonin, the number of dopaminergic neurons in some of the clusters was identical to those in non-treated control flies.

The difference between the relative efficacies of L-dopa and melatonin in reducing the toxicity of the mitochondrial complex I inhibitor in *Drosophila* is quite remarkable. While L-dopa attenuated some of the behavioral deficits associated with rotenone treatment, it failed to limit dopaminergic cell loss. Conversely, melatonin preserved normal behavior and reduced the destruction of the dopamine-containing neurons. The authors surmised that the high free radical scavenging and antioxidative activity of melatonin accounted for its protective actions against rotenone toxicity, although yet undefined mechanisms could not be excluded. On the basis of the findings, the authors suggested the use of melatonin by humans to prevent the neural oxidative damage that accompanies Parkinson disease (Coulom and Birman, 2004).

### 1-Methyl-4-phenyl-1,2,3,6-tetrahydropyridine (MPTP)

Roughly 30 years ago it was reported that young adults who unknowingly ingested MPTP developed severe neurological signs reminiscent of those seen in Parkinson disease (PD). It was immediately suspected that the drug caused damage to the dopaminergic cells of the substantia nigra which are the major neurons lost in individuals with idiopathic PD. When these individuals died and the brain was examined there was, in fact, a selective destruction of the mesencephalic dopamine-containing neurons. As tragic as these instances were, the identification of this destructive drug provided experimentalists with an agent that causes parkinson-like signs in animals. Use of MPTP has now become a model of examine the processes of PD as well as to investigate drugs that may modify the course of the disease.

When administered to animals, MPTP is taken up by astrocytes surrounding dopaminergic neurons and terminals where it is metabolized to 1-methyl-4-phenylpyridinium (MPP^+^). The latter molecule is then released from the glial cells and is taken into dopaminergic nerves via the dopamine transporter. MPP^+^ interferes with complex I of the mitochondrial ETC; this leads to cellular energy depletion and eventually to the death of dopaminergic cells.

Within a rather short interval after discovery of melatonin as an antioxidant, melatonin was used in an attempt to suppress the neural toxicity of MPTP. In this study, mice were injected with MPTP and were likewise given melatonin (Acuna-Castroviejo *et al*., 1997). Four hours later the brain of each mouse was recovered and the levels of lipid peroxidation products were found to be increased in the striatum and hippocampus as a result of MPTP administration; these increments were not observed in the mice treated with melatonin. More importantly, immunocytochemically-detected tyrosine hydroxylase activity in the dopaminergic nerve terminals in the striatum was lost as a result of the injection of MPTP; again this was prevented by melatonin. Although this study did not observe changes n the substantia nigra (likely due to the brief treatment period), the findings are consistent with a protective action of melatonin at the level of nigral dopaminergic neurons.

Shortly thereafter, a more thorough in vivo study that documented the ability of melatonin to limit damage to nigral dopaminergic neurons after MPP^+^ administration was published by Jin and co-workers (1998). Using rats, they showed that the rise in lipid peroxidation products in the substantia nigra as well as the reduction in neuronal tyrosine hydroxylase activity (the enzyme that determines dopamine synthesis) did not occur in MPP^+^-treated animals when melatonin was given concurrently. Both acutely or chronically administered melatonin attenuated the damage inflicted by MPP^+^. In mice as well, the toxicity of MPTP at the level of the substantia nigra is reduced by melatonin administration (Ortiz *et al*., 2001). The endpoints in this study were the degree of nuclear DNA fragmentation and neuronal apoptosis.

**Figure 2:**
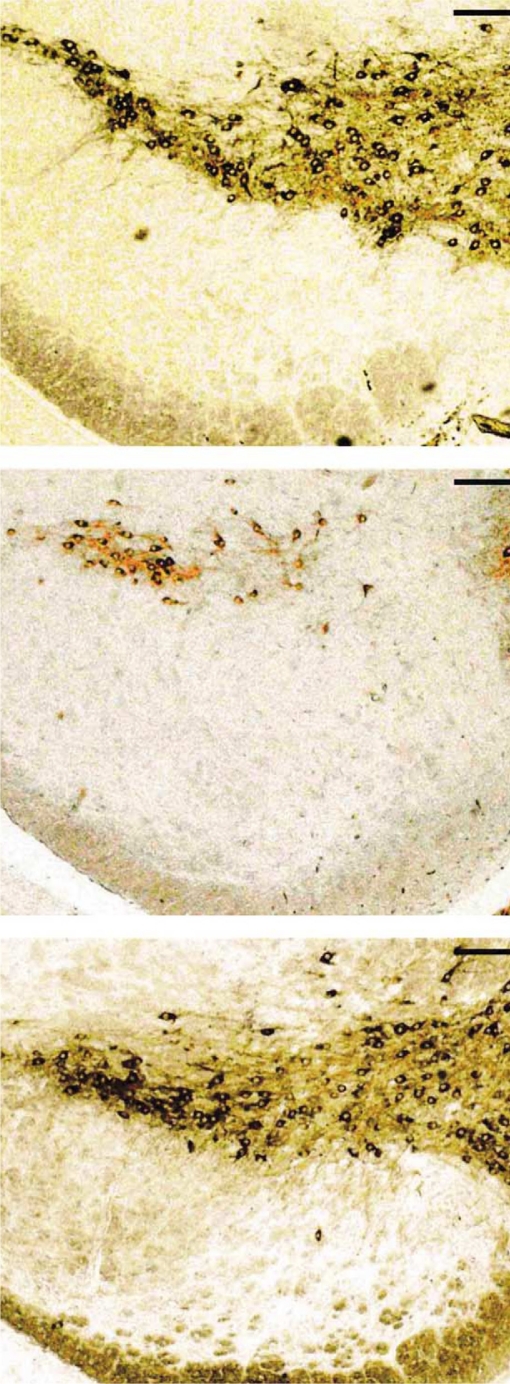
Tyrosine hydroxylase immunoreactivity of neurons in the substantia nigra of rats left untreated (top), given MPTP for 35 days (middle) or given MPTP + melatonin (bottom). In the MPTP-only treated rats, the bulk of the dopaminergic neurons were lost, a change prevented by melatonin. From Antolin and co-workers (2002).

One of the most compelling reports regarding the ability of melatonin to deflect the toxicity of MPTP at the level of the substantia nigra was published by Antolin and colleagues (Antolin *et al*., 2002). To promote a gradual reduction in the loss of dopamine-containing neurons in the substantia nigra, this group treated mice with a low daily dose of MPTP for 35 days; in half of the animals each injection of the complex I inhibitor was preceded by co-administration of melatonin. MPTP by itself caused a dramatic reduction in the number of Nissl-stained neurons and immunoreactive tyrosine hydroxylase-positive cells in the substantia nigra. In the mice given melatonin in conjunction with MPTP, the measured parameters were essentially indistinguishable from those of the control mice (Figure 2). These findings, along with others published soon thereafter (Khaldy *et al*., 2003; Chen *et al*., 2002) strongly support the idea that melatonin protects against mitochondrial complex I dysregulation induced by MPTP. That melatonin does not modify the conversion of MPTP to MPP^+^ was shown by the observation that the indoleamine did not alter the level of monoamine oxidase B (Thomas and Mohanokumar, 2004); this enzyme is responsible for the metabolism of MPTP to MPP^+^.

The most direct evidence that melatonin prevents the toxicity of MPTP/MPP^+^ at the level of the mitochondria was provided by Chen *et al*. (2005). This group measured mitochondrial DNA (mtDNA) damage as a result of MPTP administration to mice. They observed a rapid increase in immunoreactive 8-hydroxy-2-deoxyguanosine, a reliable marker of DNA damage, in mtDNA of the substantia nigra. Melatonin pre-injection, in a dose-response manner, reduced the damage. Likewise, using cultured SH-SY5Y cells Chen *et al*. (2005) also found melatonin was protective of mtDNA against MPP^+^ toxicity and, moreover, they observed that MPP^+^ time-dependently elevated mitochondrial free radical generation and a reduction in the mitochondrial membrane potential over a 24 hour period. Furthermore, 72 hours after MPP^+^ exposure, 49% of the cells had undergone apoptosis. When cells were co-incubated with a combination of MPP^+^ and melatonin, however, mitochondrial free radical generation was reduced, mitochondrial membrane potential did not collapse and cellular apoptosis was averted.

### 3-Nitropropionic acid (3-NPA)

3-NPA is a plant fungal toxin which inactivates mitochondrial succinate dehydrogenase (Coles *et al*., 1979). Succinate dehydrogenase (succinate-coenzyme Q reductase) is a key enzyme in complex II of the mitochondrial electron transport chain (Brandon *et al*., 2006). When 3-NPA is ingested by children who eat contaminated sugar cane, it causes basal ganglia lesions and marked dystonia as in Huntington's disease (HD) (Ludolph *et al*., 1991). Similarly, when this toxin is either administered systemically or if it is stereotaxically injected directly into the striatum of rodents, the neuropathological and neurobehavioral signs are similar to those seen in individuals with HD (Borlongan *et al*., 1995). Being a mitochondrial toxin, 3-NPA probably depletes cellular energy levels, which is also a deficit that likely occurs in HD (Beal, 1992).

To simulate HD, Tunez and colleagues (2004) used the mycotoxin to induce oxidative stress in the striatum and cerebral cortex of rats and tested whether melatonin would reduce the resulting damage caused by 3-NPA. 3-NPA was given at repeated intraperitoneally injected doses of 20 mg/kg BW (in dimethylsulfoxide) for 4 days while melatonin (1 mg/kg BW) was given via the same route (before, during and 4 days after mycotoxin administration). In both striatal and cerebrocortical synaptosomes, a 65–70% reduction in succinate dehydrogenase activity was measured in response to 3-NPA only; melatonin co-administration returned the activity of this mitochondrial enzyme to near control levels. Associated with the loss in succinate dehydrogenase activity and a rise in free radical generation, the levels of lipid peroxidation products and protein carbonyls were elevated; both these consequences of mycotoxin administration were highly significantly reduced by melatonin. Whether melatonin actually prevented the drop in succinate dehydrogenase activity due to mycotoxin treatment or whether it merely restored enzyme activity after 3-NPA was discontinued could not be determined from this study.

A different approach was taken by Nam *et al*. (2005) who stereotoxically unilaterally injected 3-NPA into the striatum of rats while melatonin was given 30 minutes prior to and 60 minutes after mycotoxin administration and daily for 3 days thereafter. The use of 2,3,5-triphenyltetrazolium chloride staining revealed large lesions with extensive neuronal loss in the striata that were injected with 3-NPA. Moreover, the 3-NPA lesioned rats exhibited marked ipsilateral rotational behavior in response to apomorphine. The dopamine content of the damaged striata was also diminished while malondialdehyde (an index of the peroxidation of lipids) and oxidized protein levels were elevated in the area of the lesion. Melatonin given before and after the 3-NPA injection attenuated striatal neuronal loss, limited the degree of asymmetric rotational movement, preserved dopamine levels and reduced the amount of lipid and protein oxidation.

While the results of only two studies have been published related to the use of melatonin to ameliorate 3-NPA toxicity, both reports showed conclusively that melatonin effectively counteracts the neuronal damage associated with succinate dehydrogenase poisoning. The findings are consistent with the idea that melatonin may have efficacy in the treatment of HD; this is supported by observations were melatonin was also found to be beneficial in another experimental model of this neurodegenerative condition, i.e., administration of quinolinic acid (Cabrera *et al*., 2000).

### Cyanide

Cyanide is a potent inhibitor of cellular respiration by acting on cytochrome oxidase thereby blocking electron transport. This results in reduced oxidative phosphorylation, oxygen utilization and elevated free radical generation in the mitochondria.

Yamamoto and Tang (1996) performed a series of investigations to determine whether melatonin would reverse some of the sequalae of cyanide. In a dose-dependent manner, the subcutaneous administration of potassium cyanide (6, 8, or 9 mg/kg BW) to mice caused severe tonic/clonic seizures and neural lipid peroxidation. Melatonin does-dependently reduced both the seizure incidence and lipid peroxidation in the cyanide-treated mice. The effective dose of cyanide to cause seizures in 50% of the mice, i.e., ED_50_, was elevated when melatonin was co-administered while the frequency of death in half of the animals at 24 hours (LD_50_) was increased by melatonin treatment. The reduction in cyanide-mediated neural toxicity by melatonin was assumed to be related to the free radical scavenging activity of the indoleamine.

When cultured rat cortical neurons were exposed to potassium cyanide over a range of concentrations (0.01–1.0 mM) lactate dehydrogenase efflux, indicative of cellular damage, into the culture medium was observed. Melatonin significantly reduced escape of the enzyme from the neurons and preserved their morphology (Yamamoto and Tang, 1998). These researchers also showed, both in vitro and in vivo, that the damage to rat mtDNA by cyanide is also abolished by melatonin co-treatment (Yamamoto and Mohanan, 2002). The authors assumed that the mitochondrial dysfunction caused by cyanide initiated essentially uncontrolled free radical generation leading to oxidative mutilation of the neighboring mtDNA. This being the case, melatonin's ability to limit disfiguration of mtDNA was very likely a result of the high efficiency of the indoleamine as a scavenger of reactive oxygen and reactive nitrogen species.

## Apoptosis: prevention by melatonin

One definitive endpoint of mitochondrial free radical generation is cellular apoptosis. Antioxidants that reduce the production of ROS/RNS at the mitochondrial level would be expected to limit cellular death. Using appropriate fluorescent probes, free radical production in mitochondria can be visualized along with many of the downstream events which culminate in the demise of the cell. With regard to melatonin's ability to act in mitochondria to alter the rate of free radical production, the most thorough studies are those of Jou and colleagues (Jou *et al*., 2002, 2004, 2007).

To initially observe intramitochondrial ROS, the fluorescent dye dihydrorhodamine-123, was used in astrocytes exposed to visible laser radiation. Using time-lapse confocal imaging of ROS production after subjecting astrocytes to laser irradiation, Jou *et al*. (2002) documented the dramatic increase in ROS over a 20 minutes period. The bulk of these reactants was clearly created within mitochondria. By imaging the changes at 30 sec intervals for 20 minutes, the ROS were seen to accumulate rather slowly over the first 4 minutes of laser exposure with a much more rapid increase over the last 15 minutes. Concurrent phase contrast microscopy demonstrated the presence of other apoptosis-related cellular changes, e.g., cytoplasmic blebbing, nuclear condensation, etc. Moreover, melatonin and another antioxidant, vitamin E, largely attenuated laser irradiation-induced mitochondrial ROS formation and prevented apoptosis. In their most recent investigation, Jou and co-workers (2007) documented that, even in cells that have a common deletion of mitochondrial DNA and therefore generate an excess of free radicals, melatonin is still capable of reducing oxidative damage and preventing apoptosis.

**Figure 3:**
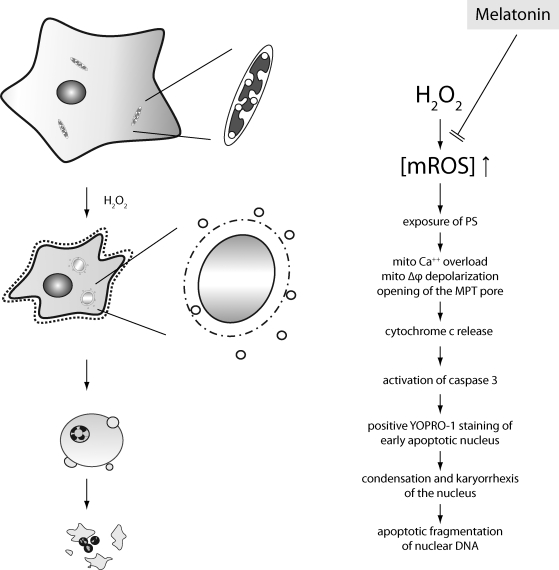
This figure summarizes the findings as uncovered by Jou *et al*. (2004). By effectively scavenging reactive oxygen species generated within mitochondria, melatonin prevents the collapse of the mitochondrial membrane potential, opening of the mitochondrial transition pore (MTP) and the downstream events that lead to cellular apoptosis. Additionally, melatonin may have direct actions on the mitochondrial transition pore that are unrelated to its scavenging activity.

In a second, more complete study, this group examined in detail many aspects of apoptosis and showed that melatonin also prevented death of cells caused by the oxidizing agent, H_2_O_2_ (Jou *et al*., 2004). With the aid of non-invasive mitochondrial-targeted fluorescent probes and time-lapse conventional, confocal, and multiphoton fluorescent imaging microscopy, the authors were able to show that melatonin effectively prevented endogenously-applied H_2_O_2_-induced mitochondrial swelling and apoptotic cell death. Moreover, melatonin reduced plasma membrane exposure of phosphatidyl serine, mitochondrial transition pore (MTP) opening, cytochrome c release, positive YOPRO-1 staining of the early apoptotic nuclei and DNA laddering. Besides inhibiting the apoptotic events initiated in astrocytes by H_2_O_2_, melatonin also reduced free radical formation and other degenerative cellular processes that resulted from the exposure of cells to either tert-butyl hydroperoxide or cumene hydroperoxide. Additionally, the protective effect of melatonin against these damaging agents was better than that provided by vitamin E (Jou *et al*., 2004). Figure 3 summarizes the processes involved in melatonin's actions in preventing free radical-mediated, mitochondrial-dependent cellular apoptosis.

The greater efficacy of melatonin in reducing observable free radical generation and apoptotic processes than vitamin E is a common observation. A number of studies have compared melatonin with classic antioxidants, e.g., glutathione, mannitol, vitamin C, vitamin E, etc., and invariably melatonin performs in a superior manner (Sofic *et al*., 2005; Qi *et al*., 2000; Martin *et al*., 2000).

According to Jou and colleagues (2004) the opening of the MTP follows free radical damage that accompanies oxidant exposure. However, other studies suggest that melatonin additionally, may have direct effects on the pore which prevent its opening and the escape of cytochrome c (Andrabi *et al*., 2004).

Besides reducing free radical-mediated, mitochondrial-dependent apoptosis, a process that does not require an interaction of melatonin with a receptor, melatonin may also have a receptor-mediated means of reducing the likelihood of apoptosis. Kilic *et al*. (2005a) examined the activation of a cytosolic signaling pathway after transient focal neural ischemia, which induces massive neuronal apoptosis, in mice. They used Western blots to analyze the phosphorylation of AKt, mitogen-activated protein (MAP) kinase/extracellular-regulated kinase (ERK)-1/2 and Jun kinase (JUN)-1/2 since they are crucial factors in reducing cellular death. On the basis of their results, however, Kilic and co-workers (2005a) concluded that phosphorylated ERK-1/2 and phosphorylated JNK-1/2 were not involved in melatonin's protection against neuronal loss after ischemia/reperfusion injury to the brain. On the other hand, melatonin markedly upregulated phosphorylated AKt suggesting that the phosphoinositol-3 kinase/Akt pathway (PI-3K) is involved in mediating melatonin's protective actions in reducing neuronal loss due to apoptosis.

A follow-up study by the same group that melatonin acts to preserve the function of the PI-3 K/Akt pathway (Kilic *et al*., 2005b); this pathway has an inhibitory effect on mitochondrial injury and caspase activation (Yoshimoto *et al*., 2001; Friguls *et al*., 2002). As a result of the activation of the PI-3K pathway, phosphorylated Akt binds to Bad, a pro-apoptotic member of the Bcl-2 family; this impedes the translocation of Bad to the mitochondria. When phosphorylated Akt levels are lowered, Bad heterodimerizes with anti-apoptotic Bcl-2 members thereby reducing their efficacy in executing their cytoprotective actions (Abe *et al*., 2004). Thus, melatonin's anti-apoptotic effects may be, at least in part, a result of its ability to preserve the activity of the PI-3K/Akt pathway. This explanation could also be used to define the findings of You *et al*. (2006) who reported the up-regulation of BcL-X_L_ and the reduction in cytochrome c release from mitochondria by melatonin in the damaged newborn rat brain.

## Concluding remarks

Mitochondria are the site of a large percentage of free radicals and related toxicants that are generated in cells. These reactive products cause damage to essential mitochondrial molecules which result in opening of the mitochondrial transition pore, release of cytochrome c and activation of the down stream events that culminate in free radical-mediated, mitochondrial-dependent apoptosis.

Since melatonin was discovered to be an indirect antioxidant and direct efficient free radical scavenger, its ability to reduce oxidative stress and to curtail cellular apoptosis has been repeatedly documented. It has also been shown that part of melatonin's ability to quell the oxidation of key molecules stems from its conversion to metabolites, i.e., cyclic 3-OHMEL, AFMK and AMK when it incapacitates free radicals and their related products.

The ability of melatonin to protect against oxygen- and nitrogen-based reactants is obvious in situations where toxins that inhibit the mitochondrial electron transport complexes are used. The mitochondrial poisons cause electron leakage with the resultant formation of large numbers of free radicals and consequentially molecular damage. This mutilation is inhibited by melatonin and its metabolites.

While melatonin readily resists mitochondrial oxidative damage and cellular apoptosis, there are many unanswered questions remaining. Some of the most noteworthy relate to the intramitochondrial concentrations of melatonin, the precise location of the indoleamine in relation to the complexes of the ETC, is it melatonin or a melatonin derivative that is the active agent in mitochondria and a definitive explanation for its high efficacy in preventing mitochondrial and cellular free radical-mediated destruction. Melatonin's very low toxicity combined with its high efficacy, however, portends its use in clinical medicine to treat conditions that are associated with elevated free radical damage, e.g., septic shock (Gitto *et al*., 2001), certain neurodegenerative conditions (Pappolla *et al*., 2003; Ishido, 2007), ischemia/reperfusion injury (Reiter *et al*., 2005), ionizing radiation (Vijayalaxmi *et al*., 2004; Manda *et al*., 2007), and toxin (Xu *et al*., 2007; Matsura *et al*., 2006) and heavy metal (Qi *et al*., 2002; El-Sokkary *et al*., 2003) exposure, among others.
